# Neurological impact of emboli during adult cardiac surgery

**DOI:** 10.1016/j.jns.2020.117006

**Published:** 2020-09-15

**Authors:** Nikil Patel, Caroline Banahan, Justyna Janus, Mark A. Horsfield, Anthony Cox, David Marshall, Jordan Colman, John Morlese, David H. Evans, Claire Hannon, Vincent Egan, Peter Garrard, James P. Hague, Emma M.L. Chung

**Affiliations:** a Department of Cardiovascular Sciences, University of Leicester, Leicester LE2 7LX, UK; b Leicester Biomedical Research Centre, Glenfield Hospital, Leicester LE3 9QP, UK; c University Hospitals of Leicester NHS Trust, Leicester LE1 5WW, UK; d School of Physical Sciences, The Open University, Walton Hall, Milton Keyns, MK7 6AA, UK; e Department of Clinical Neurosciences, St George's, University of London, London SW17 ORE, UK; f Department of Psychiatry and Applied Psychology, University of Nottingham, Nottingham NG8 1BB, UK

**Keywords:** Cerebral microbleeds, Embolization, Cardiopulmonary bypass, MRI brain imaging, Cognitive outcome

## Abstract

**Objectives:**

This study draws on advances in Doppler ultrasound bubble sizing to investigate whether high volumes of macro-bubbles entering the brain during cardiac surgery increase the risk of new cerebral microbleeds (CMBs), ischemic MR lesions, or post-operative cognitive decline (POCD).

**Methods:**

Transcranial Doppler (TCD) ultrasound recordings were analysed to estimate numbers of emboli and macrobubbles (>100 μm) entering the brain during cardiac surgery. Logistic regression was used to explore the hypothesis that emboli characteristics affect the incidence of new brain injuries identified through pre- and post-operative MRI and neuropsychological testing.

**Results:**

TCD, MRI, and neuropsychological test data were compared between 28 valve and 18 CABG patients. Although valve patients received over twice as many emboli per procedure [median: 1995 vs. 859, *p* = .004], and seven times as many macro-bubbles [median: 218 vs. 28, *p* = .001], high volumes of macrobubbles were not found to be significantly associated with new CMBs, new ischaemic lesions, or POCD. The odds of acquiring new CMBs increased by approximately 5% [95% CI: 1 to 10%] for every embolus detected in the first minute after the release of the aortic cross-clamp (AxC). Logistic regression models also confirmed previous findings that cardiopulmonary bypass time and valve surgery were significant predictors for new CMBs (both *p* = .03). Logistic regression analysis estimated an increase in the odds of acquiring new CMBs of 6% [95% CI: 1 to 12%] for every minute of bypass time over 91 mins.

**Conclusions:**

This small study provides new information about the properties and numbers of bubbles entering the brain during surgery, but found no evidence to substantiate a direct link between large numbers of macrobubbles and adverse cognitive or MR outcome.

Clinical Trial Registration URL - http://www.isrctn.com. Unique identifier: 66022965.

## Highlights

• Higher numbers of macrobubbles enter the brain during valve surgery compared to bypass graft surgery.
• Macrobubbles did not appear to be linked to new cerebral microbleeds, ischemic lesions, or cognitive decline.
• Emboli received following release of the aortic cross-clamp predicted new cerebral microbleeds.
• Other factors predicting new microbleeds included cardiopulmonary bypass duration and surgery type.

## 1 Introduction

In previous research, we showed that 76% of patients undergoing cardiac surgery with cardiopulmonary bypass (CPB) received new cerebral microbleeds (CMBs) [1], 31% of patients received new ischemic lesions [2], and 46% of patients experienced post-operative cognitive decline (POCD) [1,2]. The main aim of this study was to explore the hypothesis that bubbles entering the brain circulation during surgery might contribute to the pathogenesis of new CMBs, ischemic lesions, or POCD. Although extensive research has failed to identify any strong link between bubbles and POCD [3], the pathophysiology of CMBs is relatively unexplored. No previous studies have attempted to relate the characteristics of bubbles (size and cumulative volume of air) to neuroimaging outcomes. Evidence from human and animal models suggests that bubbles have the potential to aggravate inflammatory responses, resulting in complement activation, which reduces blood-brain barrier integrity allowing leakage of blood into the tissue [4]. In our previous study, surgery-related CMBs were found in 76% of patients following cardiac surgery [1], but the pathophysiology leading to the development of these surgery-related CMBs remains the subject of debate [5]. This sub-study explores whether bubbles received during surgery are implicated in the pathogenesis of post-operative CMBs.

Our research utilises a bubble sizing algorithm to estimate the sizes of bubbles and overall volume of air reaching the middle cerebral artery (MCA) territories during surgery [6]. The feasibility of sizing bubbles intraoperatively, and imaging findings from this study have been reported elsewhere [1,2,7]. The main aim of this sub-study was to determine whether total bubble volume, or a high incidence of macro-bubbles (defined as bubbles greater than 0.1 mm in diameter) were associated with new CMBs, ischemic lesions, or POCD.

## 2 Patients and methods

All patients undergoing coronary artery bypass grafting (CABG) and/or cardiac valve surgery at the University Hospitals of Leicester NHS Trust were eligible for inclusion. For the purposes of neuropsychological testing, patients were excluded if their first language was not English. The study was approved by the UK Clinical Research Network (ISRCTN ID: 66022965), the 10.13039/100012151University Hospitals of Leicester NHS Trust, and Derbyshire Research Ethics Committee (REC reference: 10/H0401/78), and was sponsored by the 10.13039/501100000738University of Leicester.

#### 2.1.1 Anaesthetic and surgical procedures

All patients received routine perioperative care with arterial blood pressure targets based on usual clinical practice. Non-pulsatile cardiopulmonary bypass (CPB) with a non-occlusive roller pump was used to maintain a perfusion pressure of at least 50 mmHg. The CPB circuit included a membrane oxygenator and a 40 μm arterial line filter. Surgeons employed a variety of methods, including palpation and fine-needle aspiration, to reduce emboli released during cannulation and expel air from the cardiac chambers prior to weaning from bypass. The Trendelenburg position was adopted for all patients. No aortic filters or embolus capture devices were employed. On some occasions the surgeon would gently agitate the heart to dislodge air from underneath the valve leaflets and trabeculae, bringing any air to the surface of the chambers. In patients with severe aortic calcification, distal arch cannulation was adopted to help avoid the release of solid emboli.

#### 2.1.2 Transcranial Doppler (TCD) embolus detection

To detect emboli (solid and gaseous) entering the MCA territories, intra-operative TCD monitoring was performed bilaterally using a commercially available TCD system equipped with a pair of 2 MHz transducers (DWL Doppler-BoxTM, Compumedics GmbH, Germany). Doppler signals were analysed by a blinded observer using ‘in house’ software developed in MATLAB (The Mathworks Inc., Natick, MA). Candidate embolic signals >7 dB above the average background intensity were automatically identified as peaks within the recording, and manually assessed by a highly experienced observer (CB, NP) according to recommended embolus detection criteria [8]. During analysis of very dense embolic showers it became impossible to distinguish individual emboli, and only the duration of the shower could be recorded.

#### 2.1.3 Estimating bubble size

Bubble diameters were estimated using a model based on ultrasound scattering theory*.*^6,7^ This algorithm sizes bubbles based on analysis of the backscatter intensity of the bubble relative to blood, together with information on MCA diameter gained for each individual patient from time-of-flight angiography data, see Supplemental Fig. 1 [7]. Other adjustable parameters within in the model included Doppler sample length (8–12 mm), insonation angle (assumed to be 30°), and haematocrit (recorded every 3 min during CPB). The total volume of air (*V*) entering each MCA was calculated using Eq. 1, where *r*_*i*_ is the estimated radius of each bubble:

(1) $$V=\sum_{i}\frac{4}{3}\pi r_{i}^{3}$$

Since we were unable to exclude solid emboli from our recordings, any embolic signals generated by solid emboli would have been misclassified by this algorithm as very small bubbles. This would not affect the estimated number, diameter, or volume of macrobubbles, as bubbles >100 μms generate strong backscatter signals that are not confused with particulate emboli. However, misclassification of solid emboli as small bubbles does have potential to lower estimates of median bubble diameter. The total volume of air received would also be slightly overestimated. For more details of the limitations of our sizing algorithm, please see the feasibility case studies presented by Chung et al. (2015) [7].

#### 2.1.4 MRI scans

All MRI examinations were conducted using a 3-Tesla whole body scanner (Magnetom Skyra, Siemens Medical, Erlangen, Germany). Scans were performed in the following order: 3-plane localiser; diffusion-weighted (DW) imaging; time-of-flight magnetic resonance angiography; susceptibility-weighted imaging (SWI), and fluid-attenuated inversion recovery (FLAIR). FLAIR images were obtained using a slice thickness of 3 mm with the number of slices set to cover the whole brain. Matrix size was 320 × 352, field of view was 240 mm, repetition time/echo time was 6770/108 ms, and inversion time was 2170 ms. The total imaging time was approximately 30 min. To identify new CMBs and distinguish chronic lesions from acute ischemic changes, SWI and FLAIR images were analysed independently by two blinded neuroradiologists (JM and AC).

#### 2.1.5 Identification of ischemic lesions and CMBs

FLAIR and DW images were analysed to determine the location and volume of both new and pre-existing lesions, so that chronic tissue damage could be distinguished from acute ischemic changes. Image analysis was performed using the medical image analysis package Jim (version 7, Xinapse Systems, Colchester, United Kingdom; http://www.xinapse.com) [9]. Lesions were delineated using a semiautomatic contouring technique [9]. SWI images were examined using the Brain Observer Microbleed Rating Scale to aid identification and location of CMBs [10]. CMBs were identified as focal areas of low signal on SWI, <10 mm in diameter. Symmetrical areas of basal ganglia calcification, flow voids from blood vessels, and low signal from adjacent bone were excluded. Images were assessed for the location and number of both new and pre-existing CMBs, which were delineated using the same semiautomatic contouring technique. New CMBs and FLAIR lesions were identified and characterized through registration and subtraction of pre- and postoperative images [9]. To aid visualization of the distribution of new CMBs and FLAIR lesions, postoperative images were registered to a standard MRI brain atlas [11], and new CMBs and FLAIR lesions were segmented and displayed using the atlas as a reference for the 3D display.

#### 2.1.6 Neuropsychological assessment

Neuropsychological testing was performed 1 to 2 weeks before surgery and 6 to 8 weeks postoperatively (SD, 1.25 weeks), as reported by Patel et al. 2015 [2]. Our test battery included Trail Making Tests (A and B) [12], the Grooved Pegboard Test [13], Wechsler Memory Scale-Third Edition [14], and Wechsler Abbreviated Scale of Intelligence [15]. Patients also completed a Hospital Anxiety and Depression Scale questionnaire [16]. The selected battery of tests facilitates pair-wise analysis of ‘before and after’ scores, and comparison of baseline scores with well-characterized normative data in age-matched healthy controls (analysed via *z*-score analysis). Neuropsychological assessments were performed by a trained assessor who was blinded to the bubble sizing data.

### 2.2 Statistical analysis

Individual neuropsychological test scores were converted to *z-*scores through comparison with published data describing the mean (*X*) and standard deviation (SD) of test scores measured from a population of healthy subjects, and mean (x) of test scores measured in our study, Eq. (2):

(2) $$z=\frac{x-X}{\mathrm{SD}}$$

Postoperative *z*-scores were then subtracted from preoperative *z-*scores and a significant decline in cognition was assumed if there was a drop in *z*-score of >1 SD from baseline. For timed tests (Trail Making A/B and Grooved Pegboard tests), the sign of the *z*-score was reversed, so that improved performance corresponded to a positive *z*-score.

Statistical analyses were performed in *R* (R Foundation for Statistical Computing, Vienna, Austria). Median and inter-quartile range (IQR) are presented where data was non-normally distributed unless stated otherwise. Comparisons between CABG and valve patients were performed using a *t*-test, Mann-Whitney test or Pearson's χ^2^, as appropriate. Comparisons between left and right hemispheres were made using the Chi-squared linear-by-linear association test. After summarising demographic factors and comparing differences between CABG and valve surgery, logistic regression was used to model new CMBs, new ischemic lesions, and POCD as outcome measures: emboli parameters of interest were introduced one-by-one to assess whether bubble estimates (number, size, total volume, and shower duration), or other surgery parameters (type of surgery, CPB time) were associated with cognitive decline, CMBs, or new lesions. *P*-values of less than 0.05 were considered statistically significant.

## 3 Results

A total of 103 patients consented to take part in the study. Thirty patients did not receive intra-operative TCD monitoring for logistical reasons. Of the remaining 73 TCD recordings, blinded review suggested that a further 27 sets of recordings were either incomplete, or of insufficient quality for bubble sizing. A complete flow chart summarising enrolment and data quality checks can be found in Supplemental Fig. 2. The study generated a total of 46 pairs of complete bilateral TCD recordings (18 CABG, 28 valve surgery). An analysis summary for individual patients is provided in Supplemental Table 1. All of the complete sets of TCD recordings were accompanied by pre- and post-operative MRI scans and neurocognitive assessments. The total number of patients with SWI imaging was 45.

**Fig. 1:**
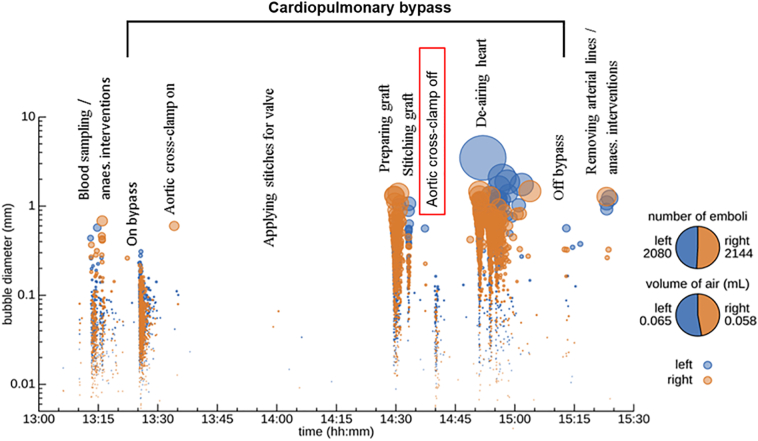
Bubble diameters estimated for a 55 year old male undergoing aortic valve replacement who had pre-existing cerebrovascular disease (total lesion volume: 1876 mm^3^) but no pre-existing CMBs. This patient exhibited cognitive decline in 2/8 tests with 10 new CMBs but no new ischemic lesions. Markers denote individual emboli (blue: left MCA events, orange: right MCA events). The vertical axis and marker size reflect estimated bubble diameter [7]. (For interpretation of the references to colour in this figure legend, the reader is referred to the web version of this article.)

Manual analysis of 115 h of TCD recordings from 46 patients identified 92,215 individual embolic signals. Total numbers of emboli received during surgery varied from 203 signals (patient 11) to 11,646 (patient 45). A representative example showing the timing and estimated sizes of bubbles received during a typical operation is shown in Fig. 1. Further patient-specific examples can be seen in our preliminary feasibility study [7].

#### 3.1.1 CABG v valve procedures

The incidence of mild to severe aortic stenosis and duration of cardiopulmonary bypass were both significantly higher in patients undergoing valve surgery than in CABG patients (*p* = .016 and *p* = .006, respectively), Table 1. In CABG patients, the prevalence of hypercholesterolemia was significantly higher than in valve patients (*p* = .007), Table 1. Otherwise, demographic factors, such as age, were similar between procedure types.

**Table 1:** Clinical and demographic characteristics of patients undergoing CABG and valve surgery.

	CABG *(n* = 18)	Valve *(n* = 28)	*p*-value
Men:women	16:2	27:1	0.552*
Age, y, mean (SD)	66 (7)	62 (9)	0.150^†^
CPB time, mins, mean (SD)	60 (22)	101 (57)	0.006^†^
Mean arterial blood pressure, mmHg, mean (SD)	64 (9)	61 (8)	0.208^†^
Mean haematocrit, %, mean (SD)	29 (4)	29 (4)	0.792^†^
Smoking, n (%)	8 (44)	13 (46)	0.895^‡^
Treated hypertension, n (%)	16 (88)	18 (64)	0.090*
Hypercholesterolemia, n (%)	18 (100)	19 (67)	0.007*
Ischemic heart disease, n (%)	11 (61)	12 (42)	0.227^‡^
Aortic stenosis (mild/severe), n (%)	5 (27)	18 (64)	0.016^‡^
Pre-existing WMD, cm^3^, mean (SD)	739 (1065)	2047 (5322)	0.217^†^
No. of old CMBs, n, mean (SD)	1 (1)	6 (9)	0.281^†^
Post-op stroke, n (%)	2 (11)	3 (16)	1.000*
Probable CAA, n (%)	2 (11)	3 (16)	1.000*

Values reported are mean (SD) or count (%) or in a ratio format. *Fisher exact-test, †t-test, ‡*χ*^*2*^ test. CMB indicates cerebral microbleeds; CPB indicates cardiopulmonary bypass; WMD indicates white matter disease; CAA indicates cerebral amyloid angiopathy.

Surgery-related CMBs were significantly more common after valve surgery (82%) than after CABG (57%), *p* = .03, Table 2. Although a higher proportion of valve patients (43%) also acquired new MRI lesions compared to 33% of CABG patients, this difference was not significant, *p* = .55, Table 2. Fewer valve patients (43%) exhibited POCD compared to CABG patients (50%), *p* = .76, but this difference was also not significant, Table 2.

**Table 2:** Comparison of imaging findings, cognitive outcome, and bubble parameters between CABG and valve surgery.

	CABG *(n* = 18)	Valve *(n* = 28)	*p*-value
New cerebral microbleeds, *n* (%)	10 (57)	23 (82)	0.031^⁎^
New FLAIR MRI lesions, *n* (%)	6 (33)	12 (43)	0.557^⁎^
Neuropsychological decline, *n* (%)	9 (50)	12 (43)	0.764^⁎^
Total emboli, n (IQR)	859 (625–1442)	1995 (1077–3127)	0.004^†^
Emboli: 1 min after removal of AxC, *n* (IQR)	6 (2–25)	39 (7–90)	0.034^†^
Macro-bubbles: ≥0.1 mm, *n* (IQR)	28 (18–108)	218 (138–505)	0.001^†^
Shower duration, seconds, *n* (IQR)	0 (0–2)	28 (0–99)	0.001^†^
Median bubble diameter, μm, *n* (IQR)	20 (10−30)	20 (20–40)	0.821^†^
Total volume of air, μL, *n* (IQR)	0.4 (0–5)	12.6 (3–26)	0.005^†^

Values reported are median (IQR) Interquartile Range (25th – 75th percentile).*Chi-squared test, †Mann-Whitney *U* test.

Patients undergoing valve procedures received more than twice as many bubbles per procedure [median: 1995 vs. 859, *p* = .004], and seven times as many macro-bubbles [median: 218 vs. 28, *p* = .001] as CABG patients. The total volume of bubbles experienced was 31 times higher for valve surgery than CABG [*p* = .005], Table 2. The median [IQR] number of emboli observed following the release of the AxC was significantly higher in patients undergoing valve surgery (39 [7–90]) compared to CABG (6 [2–25]), *p* = .03, Table 2. Median estimated bubble diameter was not significantly different between procedure types (20–30 μm), *p* = .82, Table 2.

Overall, significantly higher incidences of CMBs were observed following valve surgery, potentially linked to the release of atheromatous debris following release of the AxC, longer CPB times, and a higher number, size and volume of bubbles. Conversely, we found no evidence of any difference in the incidence of new MRI lesions and POCD between CABG and valve procedures.

#### 3.1.2 Contributors to CMBs

Patients undergoing valve surgery were significantly more likely to experience new CMBs compared to CABG, *p* = .03. Logistic regression analysis estimated an increase in the odds of acquiring new CMBs of 6% [95% CI: 1 to 12%] for every minute of bypass time over 91 mins. Patients receiving new CMBs experienced longer CPB machine times, on average, (mean, SD: 99 ± 53 mins) than patients with no new CMBs (71 ± 22 mins), *p* = .02, Table 3. The odds of acquiring new CMBs also increased by approximately 5% [95% CI: 1 to 10%] for every embolus detected in the first minute after the release of the AxC. The relationship between new CMBs and total number of emboli was of borderline significance (*p* = .06, Table 4). However, other factors relating to bubble size and volume were either non-significant, or the odds ratio included 1, which implies there may be no difference.

**Table 3:** Univariate comparison of age, total number of emboli, number of macro (>0.1 mm) bubbles, number of emboli released following removal of the aortic cross-clamp, shower duration, bubble diameter and volume of air for patients with and without new CMBs, MRI lesions, and cognitive decline.

	No new CMBs	New CMBs	*p*-value	No new MRI lesions	New MRI lesions	*p-*value	No cognitive decline	Cognitive decline	*p*-value
	*n* = 12	*n* = 33		*n* = 28	*n* = 18		*n* = 25	*n* = 21	
Age, *years* (range)	68 (46–78)	64 (41–80)	0.16*	64 (41–77)	66 (46–80)	0.57*	62 (41–77)	68 (54–80)	0.02*
Mean CPB time, mins, mean (SD)	71 (22)	99 (53)	0.02*	98 (58)	80 (23)	0.15*	94 (50)	87 (47)	0.59*
Total emboli, *n* (IQR)	1094 (818–1884)	1476 (731–2758)	0.35†	1073 (667–2070)	1761 (1087–2480)	0.13†	1357 (729–2453)	1349 (870–2096)	0.84†
Macro-bubbles: ≥0.1 mm, *n* (IQR)	157 (20–249)	169 (35–363)	0.52†	131 (26–327)	171 (112–316)	0.49†	194 (55–324)	132 (22–335)	0.33†
Emboli: 1 min after removal of AxC, *n* (IQR)	6.5 (0–38)	6.0 (0−32)	0.66†	8 (0–39)	46 (11−102)	0.04†	12 (4–68)	23 (2–75)	0.84†
Shower duration, *seconds* (IQR)	0 (0–81)	10 (0–63)	0.44†	2 (0–35)	11 (0–104)	0.45†	10 (0–52)	0 (0–82)	0.99†
Total volume of air, *μL* (IQR)	1.7 (0.2–20)	7 (1.1–21.6)	0.38†	5.3 (0.2–18.1)	6.2 (1.5–28)	0.37†	7.4 (1.5–26)	2.4 (0.2–19)	0.26†
CABG: intra-cardiac procedures	8:4	10:23	0.02‡	12:16	6:12	0.52‡	9:16	9:12	0.76‡

Values reported are mean (SD) and median (IQR) Interquartile Range (25th – 75th percentile). Age reported as mean (range). CPB; cardiopulmonary bypass, CMBs; cerebral microbleeds, CABG indicates coronary artery bypass grafting; AxC; Aortic cross-clamp. *t-test, †Mann-Whitney U test, ‡Chi-squared test.

**Table 4:** Binomial logistic regression analysis for outcomes of new CMBs, new ischemic lesions, and cognitive decline.

	New CMBs	*p*-value	New MRI lesions	*p-*value	Cognitive decline	*p*-value
	OR (95% CI)		OR (95% CI)		OR (95% CI)	
Age, *years*	0.97 (0.87–1.08)	0.57	0.97 (0.89–1.01)	0.50	1.11 (1.02–1.22)	0.01
CPB time, *mins*	1.06 (1.01–1.12)	0.03	0.96 (0.93–0.99)	0.01	1.00 (0.98–1.01)	0.99
Total emboli, *n*	1.00 (1.00–1.02)	0.06	1.00 (1.00–1.01)	0.50	1.00 (1.00–1.00)	0.49
Emboli: 1 min after removal of AxC, *n*	0.95 (0.90–0.99)	0.03	1.03 (1.00–1.10)	0.02	0.98 (0.96–1.00)	0.19

Logistic regression analyses controlling for effects of age and sex. Values presented as OR and 95% CIs. CMBs indicates cerebral microbleeds; CPB; cardiopulmonary bypass; AxC; Aortic cross-clamp.

#### 3.1.3 Contributors to ischemic lesions

The median number of emboli detected in the first minute following release of the aortic cross-clamp was significantly higher in patients with new ischemic lesions (46 emboli [IQR: 11–102]) compared to patients with no new lesions (8 emboli [IQR: 0–39], *p* = .04), Table 3. Logistic regression analysis with new lesions as the outcome revealed an increase in odds of acquiring new ischæmic lesions of 4% [95% CI: 1 to 7%] for every minute of bypass time over 91 mins (*p* = .01), Table 4. The odds of acquiring new ischemic lesions increased by approximately 3% [95% CI: 1 to 10%] for every embolus detected during the first minute following the release of the AxC (*p* = .02), Table 4.

#### 3.1.4 Contributors to cognitive decline

Advanced age was the only significant factor associated with POCD (*t*-test: *p* = .01), Table 4. This is consistent with the findings of previous research [17]. Our data provided no evidence for any relationship between emboli or bubble properties and POCD, Table 3. In our logistic regression analysis with POCD as an outcome, the odds of experiencing POCD were estimated to increase with age by 11% [95% CI: 2 to 22%] for every year of age above the age of 64, Table 4. On entering bubble properties into an exploratory logistic regression model of POCD; all odds ratio estimates were either non-significant or bounded by a narrow 95% confidence interval centred on ‘1’ suggesting additional bubbles confer no obvious increase (or decrease) in the risk of POCD, regardless of properties.

#### 3.1.5 Bubble properties

The volume of air typically received during both valve and CABG surgery were estimated to be less than 0.03 mL in all cases. Overall, only 13% of the 92,215 bubbles analysed were classified as macro-bubbles, defined as greater than 0.1 mm in diameter. Dense showers containing particularly large bubbles were observed following release of the aortic cross-clamp during valve procedures, and lasted a median duration of 28 s [IQR: 0–99]; no prolonged showers were observed during CABG, *p* = .001 (Table 2).

#### 3.1.6 Distribution of CMBs and new ischemic lesions

**Fig. 2:**
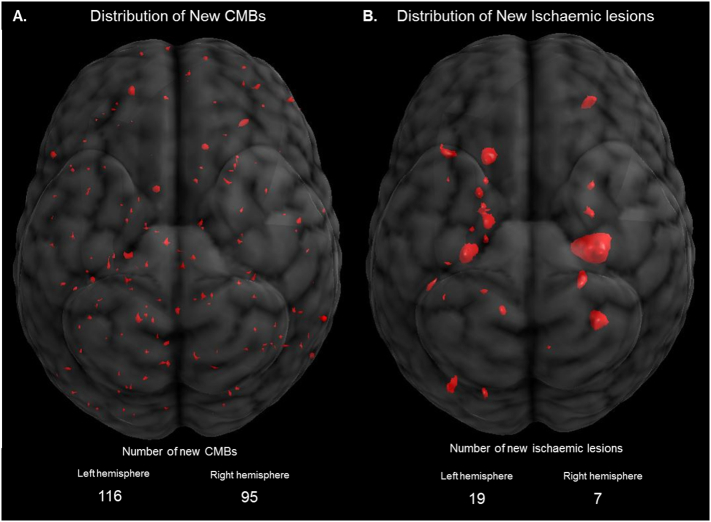
Positions of **(A)** new cerebral microbleeds, and **(B)** new MRI FLAIR lesions seen in the combined imaging data for all 46 patients. Only 4 new lesions were observed in the right hemisphere MCA territory compared to 14 in the left MCA territory; consistent with fewer emboli identified entering the right MCA in the 1 min following release of the AxC (right: 601 emboli, vs. left 1247 emboli). A similar observation was made for the CMBs, with 38 found in the right MCA territory and 58 in the left MCA territory. Estimated total numbers of bubbles and volume of air entering the MCA territories were similar for both hemispheres.

Overall, there was no significant difference in the number of CMBs observed in the left brain hemisphere (*n* = 116) compared to the right (*n* = 95), *p* = .23, Table 5, Fig. 2A. The highest numbers of CMBs were observed in the MCA territory, which is consistent with previous studies [18,19]. Similar numbers of bubbles and accumulated volume of air were detected in the left MCA (total: 49,485 (54%), median [IQR], 20 [1−10] μL) and the right MCA (total: 42,730 (46%), 20 [1–10] μL), see Table 5.

**Table 5:** Left and right hemispheric distribution of new CMBs, new ischemic lesions, and emboli entering the left and right cerebral MCA territories.

	Left cerebral territory	Right cerebral territory	*P*-value
Total emboli, *n* (IQR)	655 (387–1424)	671 (370–1130)	0.51*
Estimated volume of air, *μm, n* (IQR)	0.02 (0.001–0.01)	0.02 (0.001–0.01)	0.71*
Emboli 1 min after AxC on, *n* (IQR)	7.5 (1−23)	4.5 (0–16)	0.09*
Emboli 1 min after AxC off, *n* (IQR)	8 (2–40)	6 (0−31)	0.41*
Total MRI FLAIR ischemic lesions, *n*	**19**	**7**	0.05*
Volume of MRI FLAIR lesions, mm^3^, mean (SD)	38 (103)	50 (213)	0.72*
Middle cerebral artery (n)	15	4	0.07*
Anterior cerebral artery (n)	1	2	–
Posterior cerebral artery (n)	2	1	–
Superior cerebellar artery (n)	1	0	–
Total SWI Cerebral microbleeds	**116**	**95**	0.23*
Middle cerebral artery (n)	58	38	0.29*
Anterior cerebral artery (n)	24	32	0.60*
Posterior cerebral artery (n)	32	23	0.49*
Superior cerebellar artery (n)	2	2	–

Values reported are median (IQR) Interquartile Range (25th – 75th percentile) and mean (SD). AxC; Aortic cross-clamp, FLAIR; Fluid attenuated inversion recovery, SWI; Susceptible weighted imaging, *Chi-squared Linear-by-Linear Association test.

The total number of ischemic FLAIR lesions was more than double in the left hemisphere (*n* = 19) compared to the right (*n* = 7, *p* = .05) with the majority of the lesions in the MCA region, Table 5, Fig. 2B. The total number of emboli detected 1 min after application and release of the AxC was also higher in the left MCA compared to the right, though this was not statistically significant (AxC on: *p* = .09 and AxC off: *p* = .41), Table 5, Fig. 2B. Similarly to the distribution of CMBs, the majority of ischemic lesions were observed in the anterior/frontal regions of the brain with fewer in the posterior regions.

## 4 Discussion

This study uses a novel bubble sizing algorithm to quantify the number and sizes of bubbles released into the cerebral circulation during cardiac surgery, and provides a first exploratory analysis investigating whether bubbles increase the risk of new CMBs, ischemic lesions, or POCD. As might be expected for patients with valve disease, valve patients had a significantly higher incidence of aortic stenosis, whereas CABG patients had a higher incidence of hypercholesterolemia. Valve patients were at significantly higher risk of acquiring new CMBs, and both valve surgery and new CMBs were associated with longer CPB duration and number of emboli detected 1 min after the release of the AxC. Although valve surgery was also associated with a higher number and volume of macrobubbles, these were not significantly independently associated with new CMBs.

#### 4.1.1 Pathophysiology of new CMBs

Bubbles have potential to initiate an inflammatory response associated with activation of the complement system [4]. One hypothesis describing the pathogenesis of CMBs is that endothelial disruption, combined with other factors such as anticoagulation, blood viscosity changes, perfusion mismatch during surgery, or inflammation, can lead to localized Blood Brain Barrier leakage resulting in numerous small CMBs detected by MRI. Our bubble sizing analysis suggest a wide range of bubble diameters entering the cerebral circulation during cardiac surgery, with a median diameter of 20–40 μm.

In previous independent post-mortem examinations of the brains of patients who died shortly after cardiac surgery, Moody et al., observed highly numerous ‘sausage shaped’ arteriolar dilatations ranging between 10 and 70 μm in diameter [20]. They describe these dilations as occurring only in arterioles rather than veins [20] and report approximately 30–40 dilatations per cm^2^ in 100 μm thick sections of the basal ganglia <4 days after surgery, reducing to 1–2 dilatations per cm^2^ in patients who died a week later [20]. No dilatations were observed in patients who did not undergo CPB and no difference in the density of dilatations were identified between valve and CABG surgery. Our finding that new CMBs were associated with longer CPB duration, with no difference between procedure types, may be consistent with previous post-mortem data.

However, Moody et al. were unable to reproduce dilatations through injection of a bubble mixture to an animal bypass model [21]. Based on histological staining, they concluded that the numerous dilatations they observed in brain tissue must be due to lipid emboli [22]. Unfortunately, our embolus analysis techniques are currently unable to distinguish lipid from air emboli, and therefore a link between lipid emboli, small arteriolar dilatations and CMBs has yet to be clearly established.

One explanation for the pathogenesis of surgery-related CMBs is that bubbles last sufficiently long in the brain's circulation to lead to endothelial disruption, which when combined with other factors, such as anticoagulation, blood viscosity changes, perfusion mismatch during surgery, accumulation of lipids, and inflammation, could lead to localized Blood Brain Barrier leakage resulting in widespread small CMBs, some of which become large enough to be visible in MRI scans. It is important to establish whether the pathogenesis of surgery-related CMBs differs from that of chronic CMBs related to cerebral amyloid angiopathy, since the presence of CMBs is considered to be a contraindication for prescription of anticoagulant medication. Further research would be of interest to confirm whether circulating bubbles or lipids provide a plausible explanation for the pathogenesis of surgery-related CMBs.

#### 4.1.2 Causes of new ischemic lesions

In the current study, CPB time and emboli detected 1 min after AxC removal were significant predictors for new ischemic lesions. Focal cerebral ischemic injury is not a rare consequence of cardiac surgery, and new lesions were found in 39% of our cohort following surgery. The association with AxC emboli is consistent with the release of atheromatous debris, as described in several previous research studies [23]. Although patients with new MRI lesions tended to receive a larger volume of air, higher number of macro-bubbles, and experience longer shower durations these trends did not reach statistical significance. Our study was therefore unable to rule out air emboli as a contributory factor for new ischemic lesions. The number of emboli detected 1 min after AxC was higher in the left MCA compared to the right which was consistent with the greater numbers of new ischemic lesions observed in the left hemisphere. Although the number of new lesions was higher in the left hemisphere, the volume of new lesions was higher in the right hemisphere. This distribution in number and volume of new lesions is consistent with a cardio-embolic pathogenesis with larger emboli tending to disproportionately favour major vessels [24].

#### 4.1.3 Causes of post-operative cognitive decline

Overall, we found a 45% incidence of POCD assessed 7 weeks post-operatively, which is similar to levels reported elsewhere [[25], [26], [27]]. Our exploratory logistic regression analysis confirmed previous reports of a link between advanced age and risk of POCD [[27], [28], [29]], but found no evidence to support the hypothesis that bubbles, or any other surgical factor such as procedure type or duration of CPB, contribute to an increased risk of developing POCD.

#### 4.1.4 Limitations of this study

A major limitation of our study is its small sample size. We initially set out to detect bubbles in over 100 patients, which would have offered sufficient statistical power to identify a ~ 20% difference between groups. Technical problems with our TCD recordings vastly reduced our sample size, see Supplemental Fig. 2. A study powered to confirm the 10% difference in the incidence of POCD and MRI lesions between procedure types identified in our cohort would require around 500 patients.

A technical limitation is that no technology currently exists to reliably distinguish between thrombus, lipid, and gaseous emboli [30]; so any weak embolic signals from lipids or thrombus particles would have been misclassified by our sizing algorithm as small bubbles. Misclassification of thrombus and lipid emboli as small bubbles will have influenced our bubble parameter estimates, and limits our ability to draw firm conclusions relating to the separate contributions of thrombus, lipid and gas emboli to cognitive and MRI outcomes.

The presence of dense showers of bubbles during valve procedures also made it difficult to detect and size individual emboli during some parts of the procedure. The total number of emboli and volume of air entering the circulation reported during valve procedures was therefore likely to be underestimated. There are several potential sources of inaccuracy in our estimates of bubble size, which are particularly sensitive to uncertainties in MCA diameter. To reduce these errors, every effort was made to accurately measure MCA diameter and to optimise the TCD signal.

## 5 Conclusions

The main novelty of this study lies in our quantification of the total number and sizes of bubbles received throughout cardiac surgery. We used this to explore whether large bubbles or cumulative volume of air, might be influential in the development of new CMBs, ischemic MR lesions, or POCD. Although our study does provide new information about the properties and numbers of bubbles entering the brain during surgery, we found no evidence of a link between large numbers of macrobubbles and adverse cognitive or MR outcome.

Valve patients received more CMBs; averaging 6.6 new CMBs per patient compared to 1.2 new CMBs following CABG. Valve patients also received a significantly higher estimated volume of bubbles, and had longer CPB times than patients undergoing CABG. Patients with higher numbers of emboli detected in the first minute following release of the aortic cross-clamp were more likely to receive new ischemic lesions, explained by the release of atheromatous debris. Our study confirmed previous findings that POCD tends to occur in elderly subjects. POCD did not appear to be significantly linked to the procedure types studied here (valve replacement/repair or CABG) or operative parameters. Based on these hypothesis-generating findings, we propose that bubbles may play a role in the pathogenesis of small arteriolar dilatations and surgery-related CMBs.

## Appendix A Supplementary data

Supplementary matrial Image 1

## References

[1] Patel N., Banahan C., Janus J., Horsfield M.A., Cox A., Li X. (2019). Perioperative cerebral microbleeds after adult cardiac surgery. Stroke.

[2] Patel N., Horsfield M.A., Banahan C., Janus J., Masters K., Morlese J. (2015). Impact of perioperative infarcts after cardiac surgery. Stroke.

[3] Patel N., Minhas J.S., Chung E.M. (2016). Intraoperative embolization and cognitive decline after cardiac surgery: a systematic review. Semin Cardiothorac Vasc Anesth.

[4] Barak M., Katz Y. (2005). Microbubbles: pathophysiology and clinical implications. Chest.

[5] Patel N., Hannon C., Chung E.M.L. (2019). Response by Patel et al to letter regarding article, “perioperative cerebral microbleeds after adult cardiac surgery”. Stroke.

[6] Banahan C., Hague J.P., Evans D.H., Patel R., Ramnarine K.V., Chung E.M. (2012). Sizing gaseous emboli using Doppler embolic signal intensity. Ultrasound Med. Biol..

[7] Chung E.M., Banahan C., Patel N., Janus J., Marshall D., Horsfield M.A. (2015). Size distribution of air bubbles entering the brain during cardiac surgery. PLoS One.

[8] Ringelstein E.B., Droste D.W., Babikian V.L., Evans D.H., Grosset D.G., Kaps M. (1998). Consensus on microembolus detection by TCD. International consensus group on microembolus detection. Stroke.

[9] Patel N., Horsfield M.A., Banahan C., Thomas A.G., Nath M., Nath J. (2017). Detection of Focal Longitudinal Changes in the Brain by Subtraction of MR Images. AJNR Am. J. Neuroradiol..

[10] Cordonnier C., Potter G.M., Jackson C.A., Doubal F., Keir S., Sudlow C.L. (2009). Improving interrater agreement about brain microbleeds: development of the brain observer MicroBleed scale (BOMBS). Stroke.

[11] Mazziotta J., Toga A., Evans A., Fox P., Lancaster J., Zilles K. (2001). A probabilistic atlas and reference system for the human brain: international consortium for brain mapping (ICBM). Philos Trans. R Soc Lond B Biol. Sci..

[12] Reitan R.M. (1955). The relation of the trail making test to organic brain damage. J. Consult. Psychol..

[13] Klove H. (1963). Clinical neuropsychology. Med. Clin. North Am..

[14] Lo A.H., Humphreys M., Byrne G.J., Pachana N.A. (2012). Test-retest reliability and practice effects of the Wechsler memory scale-III. J. Neuropsychol..

[15] Harman-Smith Y.E., Mathias J.L., Bowden S.C., Rosenfeld J.V., Bigler E.D. (2013). Wechsler adult intelligence scale-third edition profiles and their relationship to self-reported outcome following traumatic brain injury. J. Clin. Exp. Neuropsychol..

[16] Zigmond A.S., Snaith R.P. (1983). The hospital anxiety and depression scale. Acta Psychiatr. Scand..

[17] Sauer A.C., Veldhuijzen D.S., Ottens T.H., Slooter A.J.C., Kalkman C.J., van Dijk D. (2017). Association between delirium and cognitive change after cardiac surgery. Br. J. Anaesth..

[18] Mesker D.J., Poels M.M., Ikram M.A., Vernooij M.W., Hofman A., Vrooman H.A. (2011). Lobar distribution of cerebral microbleeds: the Rotterdam scan study. Arch. Neurol..

[19] Jeon S.B., Lee J.W., Kim S.J., Chung C.H., Kwon S.U., Choi C.G. (2010). New cerebral lesions on T2*-weighted gradient-echo imaging after cardiac valve surgery. Cerebrovasc. Dis..

[20] Moody D.M., Brown W.R., Challa V.R., Stump D.A., Reboussin D.M., Legault C. (1995). Brain microemboli associated with cardiopulmonary bypass: a histologic and magnetic resonance imaging study. Ann. Thorac. Surg..

[21] Moody D.M., Bell M.A., Challa V.R., Johnston W.E., Prough D.S. (1990). Brain microemboli during cardiac surgery or aortography. Ann. Neurol..

[22] Stump D.A. (2007). Cannulae and cell saver design: do they make a difference?. J. Extra Corpor Technol..

[23] Banbury M.K., Kouchoukos N.T., Allen K.B., Slaughter M.S., Weissman N.J., Berry G.J. (2003). Emboli capture using the Embol-X intraaortic filter in cardiac surgery: a multicentered randomized trial of 1,289 patients. Ann. Thorac. Surg..

[24] Chung E.M., Hague J.P., Chanrion M.A., Ramnarine K.V., Katsogridakis E., Evans D.H. (2010). Embolus trajectory through a physical replica of the major cerebral arteries. Stroke.

[25] Knipp S.C., Matatko N., Wilhelm H., Schlamann M., Massoudy P., Forsting M. (2004). Evaluation of brain injury after coronary artery bypass grafting. A prospective study using neuropsychological assessment and diffusion-weighted magnetic resonance imaging. Eur. J. Cardiothorac. Surg..

[26] Gottesman R.F., Hillis A.E., Grega M.A., Borowicz L.M., Selnes O.A., Baumgartner W.A. (2007). Early postoperative cognitive dysfunction and blood pressure during coronary artery bypass graft operation. Arch Neurol..

[27] Newman M.F., Kirchner J.L., Phillips-Bute B., Gaver V., Grocott H., Jones R.H. (2001). Longitudinal assessment of neurocognitive function after coronary-artery bypass surgery. N. Engl. J. Med..

[28] Kok WF, Koerts J, Tucha O, Scheeren TW, Absalom AR. Neuronal damage biomarkers in the identification of patients at risk of long-term postoperative cognitive dysfunction after cardiac surgery. Anaesthesia. 2017;72:359–369. 10.1111/anae.13712 27987229

[29] Bartels K., McDonagh D.L., Newman M.F., Mathew J.P. (2013). Neurocognitive outcomes after cardiac surgery. Curr. Opin. Anaesthesiol..

[30] Banahan C., Rogerson Z., Rousseau C., Ramnarine K.V., Evans D.H., Chung E.M. (2014). An in vitro comparison of embolus differentiation techniques for clinically significant macroemboli: dual-frequency technique versus frequency modulation method. Ultrasound Med. Biol..

