# Supplementary material for: Neurological impact of emboli during adult cardiac surgery
Source: J Neurol Sci. 2020 Sep 15;416:117006. doi: 10.1016/j.jns.2020.117006 (PMC7718579; doi:10.1016/j.jns.2020.117006)
Supplement: Supplementary file 1 — Supplementary matrial [file mmc1.pdf]

Supplemental Figure 1.

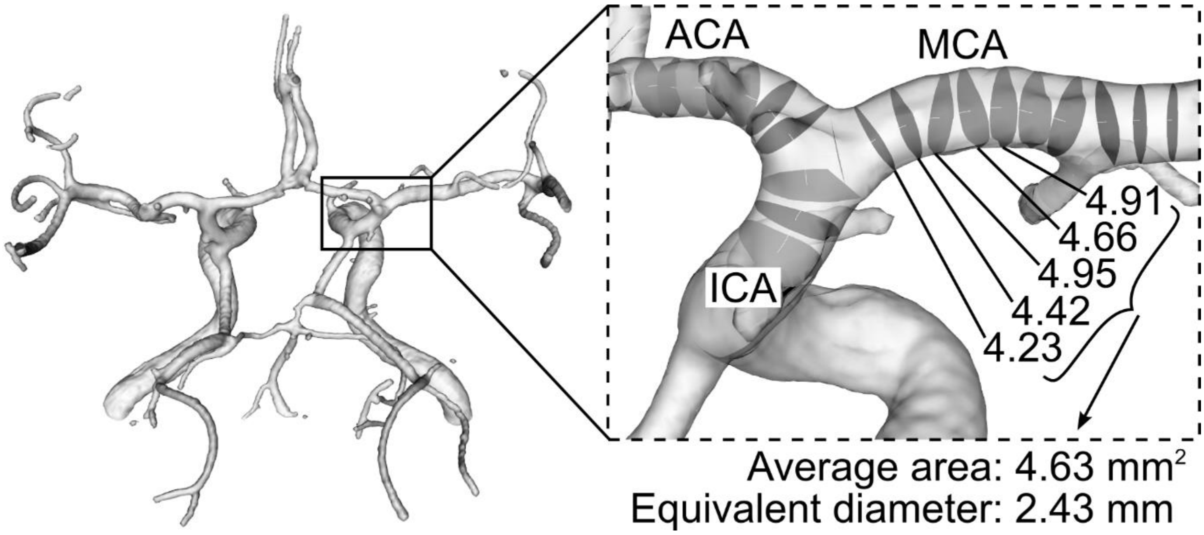

Supplemental Figure 1. 3-D reconstruction of the circle of Willis for estimating average MCA diameter. To improve the accuracy of the bubble sizing algorithm, patient specific measurements of the MCA diameter were obtained by 3D reconstruction of the circle of Willis for each patient using time-of-flight magnetic resonance (MR) angiography (Magnetom Skyra, Siemens Medical, Erlangen, Germany). The Vascular Modelling Toolkit (Antiga, 2008) was used for analysis. Images were segmented using level sets at a constant threshold for all patients. Mean diameter was calculated by averaging 5 cross sections separated by distances equal to the vessel radius, starting at two vessel radii from the internal carotid artery bifurcation. Sections on poorly resolved areas were discarded. Diameter error was estimated using the standard deviation of area measurements. Methods were chosen to provide robust objective measurement since errors in MCA diameter can modify volume estimates by up to a factor of 3 for small bubbles.

Supplemental Figure 2.

Supplemental Figure 2. A complete flow chart summarising enrolment and data quality checks

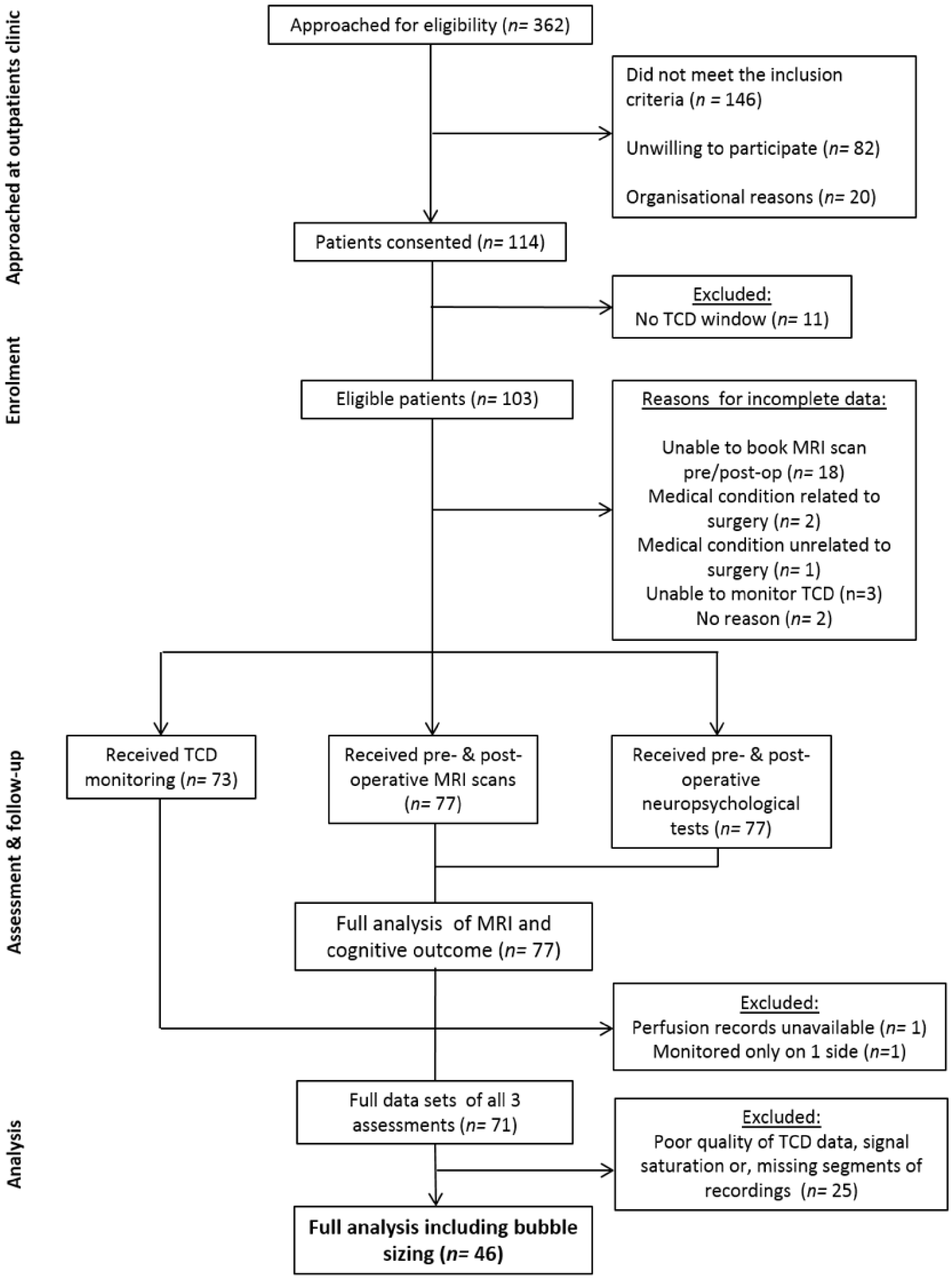

Supplemental Table 1.

Supplemental table 1. Detailed summary of age, sex, type of procedure, cardiopulmonary bypass (CPB) duration, aortic cross-clamp (AxC) duration, total number of emboli, length of dense embolic showers (curtain) total volume of air, and outcome of neurocognitive testing for all 46 patients.

Patient	Sex/Age	Surgical procedure	CPB (mins)	AxC (mins)	Total number of emboli			Length of curtain (seconds)	Estimated total volume of air (µl)	Number of tests exhibiting cognitive decline	New MRI lesions No./volume (mm^3)	New CMBs
					Left	Right	Total					
**1**	M/61	CABG	77	55	784	549	1333	0	0.86	5	1/17	-
**2**	M/60	CABG	73	39	409	383	792	0	0.27	0	0	3
**3**	M/65	CABG	48	34	636	380	1016	0	0.06	6	0	0
**4***	F/71	CABG	108	40	674	843	1517	0	36.83	0	1/1383	4
**5**	M/71	CABG	76	48	196	175	371	0	0.19	4	0	0
**6**	M/76	CABG	86	45	950	601	1551	37	22.46	1	0	0
**7**	M/68	CABG	54	24	286	364	650	0	0.12	0	00	0
**8**	M/69	CABG	58	43	284	317	601	0	2.29	4	1/51	1
**9**	M/63	CABG	66	47	1423	779	2202	0	0.04	0	1/63	1
**10***	M/66	CABG	95	44	267	191	458	0	1.57	1	1/175	2
**11**	M/77	CABG	75	47	154	49	203	4	7.24	0	0	1
**12**	M/57	CABG	62	50	253	606	859	10	53.61	0	0	3
**13**	M/77	CABG	54	25	503	367	870	0	0.43	8	0	0
**14**	M/72	CABG	53	33	1114	766	1880	0	0.12	7	0	2
**15**	M/56	CABG	74	56	398	268	666	0	0.90	0	0	1
**16**	M/53	CABG	62	44	270	534	804	0	0.04	0	0	2
**17**	F/62	CABG	35	13	354	777	1131	0	0.18	7	0	0
**18**	M/76	CABG	122	58	1559	1116	2675	110	10.63	0	2/49	0
**19**	M/62	AVR	80	42	1429	1319	2748	18	15.77	0	0	5
**20**	F/71	AVR	66	45	564	736	1300	102	15.18	7	1/309	5
**21**	M/59	AVR	89	58	1069	936	2005	10	1.35	6	1/28	4
**22**	M/46	AVR	80	44	568	489	1057	0	2.61	0	1/26	0

**23**	M/80	AVR	65	42	2107	1378	3485	64	0.05	7	2/167	8
**24***	M/50	AVR	63	35	1308	922	2230	12	7.62	0	2/389	8
**25**	M/59	AVR	71	46	1055	940	1995	96	4.62	0	0	0
**26**	M/65	AVR	120	80	617	371	988	86	27.56	0	1/15	2
**27**	M/78	AVR	62	36	434	367	801	110	23.88	5	0	0
**28**	M/54	AVR	87	63	164	458	622	20	0.04	0	0	1
**29**	M/72	AVR	92	64	757	485	1242	18	2.16	0	0	2
**30**	M/68	AVR	87	65	1244	1172	2416	0	216.00	6	1/67	0
**31**	M/54	AVR	112	85	1435	41	1476	40	0.12	7,8	0	1
**32**	M/63	AVR	72	45	911	1185	2096	28	18.86	6	0	3
**33**	M/71	MVR	75	50	502	596	1098	14	21.30	0	2/224	4
**34**	M/41	MVR	153	105	1656	2616	4272	74	30.45	0	0	23
**35**	M/64	MVR	100	77	480	886	1366	62	2.59	2,8	1/5	3
**36***	M/64	MVR	64	46	3130	1449	4579	140	145.51	0	1/81	11
**37***	M/57	MVR	44	34	550	1465	2015	2178	28.73	0	5/979	1
**38**	M/64	AVR/CABG	80	50	509	88	597	0	12.56	0	0	1
**39**	M/55	AVR/CABG	102	71	2080	2144	4224	130	122.89	2,8	0	10
**40**	M/69	AVR/CABG	111	95	1813	956	2769	180	2.62	8	0	1
**41**	M/65	AVR/CABG	219	72	191	391	582	0	7.07	0	0	11
**42**	M/66	MVR/CABG	110	80	349	322	671	0	15.80	2	0	16
**43**	M/74	MVR/CABG	53	55	2785	3691	6476	0	4.77	0	1/37	7
**44**	M/56	MVR/CABG	119	80	730	742	1472	0	24.40	0	0	5
**45**	M/72	MVR/TVR	271	225	6691	4955	11646	0	6.03	8	0	8
**46**	M/61	MVR/TVR/CABG	254	234	3843	2565	6408	30	10.70	0	0	43

Patients marked with * had perioperative stroke diagnosed clinically. CABG indicates coronary artery bypass graft; AVR, aortic valve replacement; MVR, mitral valve replacement; TVR, tricuspid valve replacement. Cognitive test 1 indicates immediate memory; 2, Delayed memory; 3, Verbal intelligence; 4, Performance intelligence; 5, Trail Making Test A; 6, Trail Making Test B; 7, Grooved Pegboard (dominant); 8, Grooved Pegboard (non-dominant).
